# Groundwater arsenic content related to the sedimentology and stratigraphy of the Red River delta, Vietnam

**DOI:** 10.1016/j.scitotenv.2021.152641

**Published:** 2022-03-25

**Authors:** Jolanta Kazmierczak, Dieke Postma, Trung Dang, Hoan Van Hoang, Flemming Larsen, Andreas Elmelund Hass, Andreas Hvam Hoffmann, Rasmus Fensholt, Nhan Quy Pham, Rasmus Jakobsen

**Affiliations:** a Geological Survey of Denmark and Greenland, Department of Geochemistry, Øster Voldgade 10, 1350 Copenhagen, Denmark; b Hanoi University of Science and Technology, Department of Geology, Hanoi, Vietnam; c Hanoi University of Mining and Geology, Department of Hydrology, Hanoi, Vietnam; d University of Copenhagen, Department of Geosciences and Natural Resource Management, Copenhagen, Denmark

**Keywords:** Groundwater arsenic contamination, Remote sensing, Sedimentary floodplain structure, Hydrogeological processes, Arsenic hazard maps

## Abstract

Arsenic (As) is highly toxic and over 100 million people living on the floodplains of Asia are exposed to excessive groundwater As. A very large spatial variability over small distances has been observed in the groundwater As concentrations. Advances in the prediction of the As distribution in aquifers would support drinking water management. The application of remote sensing of geomorphic paleo river features combined with geological, geophysical and archeological data and available groundwater As measurements may be used to predict groundwater As levels in rural areas, as shown by the example from the Red River delta, Vietnam. Groundwater in sediments deposited in the marine environment is low in As, probably due to the precipitation of As in sulfide minerals under anoxic conditions. Groundwater As levels in freshwater alluvial deposits in undisturbed floodplain areas are slightly increased and the highest As concentrations are associated with meander belts. The meander belts remain clearly visible in remote sensing and may well reflect the youngest preserved alluvial sediments. High As levels in the meander belt aquifers are probably related to the availability of highly reactive organic matter and consequent reduction of iron oxyhydroxides and As release. Furthermore, given similar hydrogeological conditions, the extent of flushing of As from the youngest alluvial sands is limited compared to the older Pleistocene sands. Even within abandoned meander belts a high spatial variability of As concentrations was observed. The younger channel belts (<1 ka BP) and old Holocene aquifers below undisturbed floodplain environments deposited during a period with high sea level host groundwater enriched in As. Low As groundwater is found in sandy channel belts deposited during the regression of the sea and in Pleistocene islands preserved within the floodplain. The decisive influence of the depositional environment of the aquifer sediments on groundwater As content is revealed.

## Highlights

• Arsenic (As) hazard maps improved by Remote Sensing (RS) and archeological data.
• Sedimentary structure of a delta plain interpreted from RS and ground based methods.
• Significant insight into groundwater As provided from detailed geology.
• Extent of low As groundwater in the marine deposits derived from RS.
• High As groundwater associated with meander belts and thick clay layers.

## Graphical abstract

**Figure:**
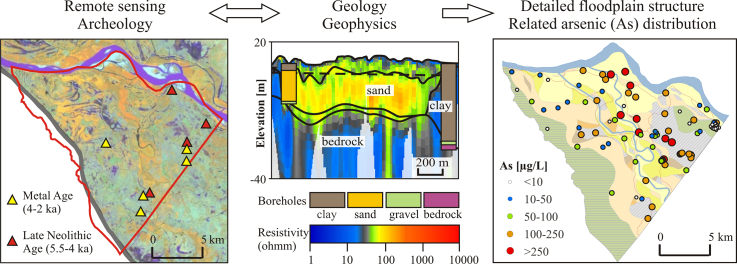

## 1 Introduction

Groundwater arsenic (As) contamination is a common problem in Asia (Fendorf et al., 2010; Winkel et al., 2011; Zhang et al., 2012). Arsenic is highly toxic (Argos et al., 2010; Chen et al., 2011; Rahman et al., 2014) and more than 100 million people living on the floodplains of south and southeast Asia drink groundwater containing toxic levels of As (Ravenscroft et al., 2009). On the Red River floodplain alone, it is estimated that 3 million people have excessive As in their drinking water (Winkel et al., 2011).

Arsenic is a natural geogenic contaminant released from Holocene aquifer sediments into groundwater (Postma et al., 2007; Fendorf et al., 2010; Postma et al., 2010, Postma et al., 2012, Postma et al., 2016; Sø et al., 2018a). One of the major problems in managing this contamination problem is a very large spatial variation in groundwater As content over even small distances (van Geen et al., 2003; Papacostas et al., 2008; Winkel et al., 2011) which requires surveys on the As content of groundwater in every well. The origin of the variation remains poorly understood (van Geen et al., 2003; Winkel et al., 2011). The As hazard maps produced to date use a statistical analysis of geological and surface variables to predict areas potentially contaminated with As (Winkel et al., 2011; Zhang et al., 2012), but are unable to resolve small scale variability (Winkel et al., 2011). A better understanding of the processes controlling this variability could be used to make more reliable predictions of groundwater As content with a high spatial resolution.

Arsenic is transported by rivers with sediment particles and is particularly associated with iron oxyhydroxides (McArthur et al., 2001; Dowling et al., 2002; Harvey et al., 2002; Swartz et al., 2004; Postma et al., 2016). The oxidized form of As (As(V)) is contained in iron oxyhydroxides, while the reduced form (As(III)) is either present in the groundwater, adsorbed on the surface layer of iron oxyhydroxides or incorporated in reduced minerals such as iron sulfides (Postma et al., 2016). Dissolved As is predominantly present as As(III) with uncharged H_3_AsO_3_ being the main species (Postma et al., 2007). As-rich iron oxyhydroxides become deposited in Asian deltas along with organic matter that is particularly abundant in fine-grained floodplain, oxbow lake and wetland deposits (McArthur et al., 2001; Dowling et al., 2002; Tanabe et al., 2006; Kocar et al., 2014; Donselaar et al., 2017), which are reworked in delta front deposits by waves and tides. Once the sediment becomes incorporated in the groundwater zone, it is no longer in contact with atmospheric oxygen and anoxic conditions often develop within a few meters below the groundwater table because of the degradation of sedimentary organic matter (Dowling et al., 2002; Swartz et al., 2004; Postma et al., 2010; Kocar et al., 2014). Iron oxyhydroxides function as important electron acceptors for organic matter oxidation in the anoxic groundwater zone and the resulting reductive dissolution of the iron oxyhydroxides causes the release of iron (Fe(II)) and As into groundwater (McArthur et al., 2001; Dowling et al., 2002; Harvey et al., 2002; Swartz et al., 2004; Postma et al., 2016). Groundwater As contents may become further modified by sorption and precipitation of As in secondary phases (Itai et al., 2010; Postma et al., 2016; Nguyen et al., 2014; Sø et al., 2018b).

The groundwater As concentration depends on sediment age, the extent of flushing (Postma et al., 2012, Postma et al., 2016; Sø et al., 2018a) and the availability of reactive organic carbon in sediments or water bodies above and inside the aquifers (Das et al., 2021). The As content is further influenced by constraints on the groundwater flow, e.g. due to the presence of silt and clay layers limiting aquifer flushing (Donselaar et al., 2017; Hoque et al., 2017; Sø et al., 2018a) and by groundwater extraction (Donselaar et al., 2017).

An explicit relation between geomorphology and As distribution exists (Papacostas et al., 2008; Quicksall et al., 2008; Weinman et al., 2008; Sahu and Saha, 2015; Das et al., 2021). High As groundwater is typically found below low lying active flood plains and cut-offs of paleochannels filled with mud enriched in organic matter (Nath et al., 2010; Sahu and Saha, 2015; Donselaar et al., 2017; Das et al., 2021) and in paleochannels filled with younger Holecene sand and gravel (Postma et al., 2012; Donselaar et al., 2017). Low As groundwater typically occurs within paleo-levees and point bar platforms (Papacostas et al., 2008; Weinman et al., 2008; Nath et al., 2010; Sahu and Saha, 2015; Das et al., 2021).

River features at the local scale are often delineated using satellite images and digital elevation models supported by geological borehole information (Papacostas et al., 2008; Weinman et al., 2008; Sahu and Saha, 2015; Das et al., 2021). Satellite images and topography are also used in geostatistical analysis to produce As hazard maps at the regional scale (Lado et al., 2008; Winkel et al., 2008; Winkel et al., 2011; Zhang et al., 2012; Lado et al., 2013; Sovann and Polya, 2014). However, this type of geostatistical analysis does not provide detailed information on the extent of paleo river features.

Electrical resistivity data and gamma logging may further support the interpretation of the sedimentary structure of complex alluvial systems (Weinman et al., 2008; Nath et al., 2010; Tran et al., 2012; Cao et al., 2018; Chandra et al., 2019), but are rarely applied in the context of As hazard prediction. Electrical resistivity and gamma logging enable delineating the extent of sand and clay layers (Nath et al., 2010; Cao et al., 2018; Chandra et al., 2019). Gamma radiation detected in a borehole log originates from potassium-40 and daughter products of uranium and thorium decay series that are common in clay minerals (Keys, 1990). Thus, high gamma radiation indicates clay layers. Additionally, clay minerals, and thus clay and silt layers, have a low resistivity in contrast to the highly resistive sand and gravel deposits (Keys, 1990). This enables the mapping of fine-grained floodplain and oxbow lake deposits and coarse-grained channel belt sediments. Electrical resistivity has been applied in the context of As contamination of aquifers in Ganges delta, India (Nath et al., 2010; Chandra et al., 2019), where As enriched aquifers below mud layers topping the sedimentary sequence were delineated using electrical resistivity tomography (Nath et al., 2010). Paleochannels filled with sand and gravel creating preferential flow paths for As contaminated groundwater were mapped using surface signatures and airborne electromagnetics (Chandra et al., 2019). On the Yellow River plain, China, the sum of the clay-sand ratio and the number of clay layers were interpreted from gamma borehole logs and used to calculate a “swing intensity index” that was found to be proportional to the groundwater As concentration (Cao et al., 2018).

Probability maps for As, produced based on statistical analysis that includes three-dimensional (3D) geological information are more accurate than those based on solely surface variables, such as soil and elevation data (Winkel et al., 2011). However, 3D geological models interpolated from scarce borehole data can only capture the complex floodplain structures to a limited extent. More detailed regional geological models of the shallow subsurface in floodplain environments could improve the predictability of As hazards. However, the collection of extensive geological data is expensive. Instead, shallow alluvial sedimentary structures can, at the local scale, be predicted based on satellite images (Papacostas et al., 2008; Sahu and Saha, 2015) and geophysical data (Nath et al., 2010; Cao et al., 2018; Chandra et al., 2019). These datasets obtained with lower costs, e.g. satellite images and geophysics, should also be utilized in detailed geological modeling and prediction of groundwater As distribution at regional scales.

The objectives of the study were: (1) to relate groundwater As concentrations to sedimentary structure at different scales in areas of the Red River floodplain, Vietnam with scarce geological and hydrogeochemical data, (2) to reconstruct the geological evolution of the Red River floodplain and understand how it has influenced As concentrations in groundwater, and (3) to demonstrate the usefulness of remote sensing, especially satellite images, in predicting the groundwater As distribution at the delta and regional scale.

## 2 Study area

**Fig. 1:**
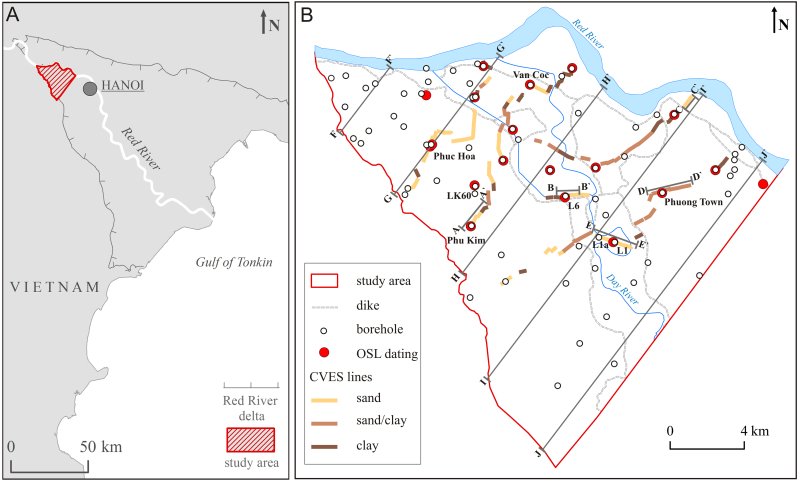
(A) Location of the Red River delta. (B) Field installations and CVES (Continuous Vertical Electrical Sounding). The colour of the CVES lines indicates dominating sediment type along the line. Cross-section lines A-A´ to *E*-E´ are the CVES data presented in Fig. 7. Cross-section lines F-F´ to J-J´ are geological cross-sections shown in Fig. 8. (For interpretation of the references to colour in this figure legend, the reader is referred to the web version of this article.)

The study was conducted in the Red River delta, Vietnam (Fig. 1A) with a focus on its proximal NW part (Fig. 1) where the floodplain stratigraphy and sedimentology have not been studied in detail before and where groundwater flow paths are not disturbed by water abstraction of Hanoi city (Bui et al., 2012). The delta has an area of 10.3 × 10^3^ km^2^ (Tanabe et al., 2006) and is covered with a dense network of irrigation channels and dikes constructed since c.a. 1 ka Before Present – BP (Anh and Shannon, 2010).

The climate in the Red River delta is characterized by a dry season from November to April and monsoon season from May to October (Larsen et al., 2008). The median annual precipitation in the Red River delta in the period 2007–2016 was 1.5 m, and evaporation 0.7 m. About 70% of precipitation occurs in the monsoon season with a daily rainfall >0.1 m during extreme events (Larsen et al., 2008).

The Red River delta comprises four aquifers: (1) a lower Pleistocene aquifer consisting of coarse sediments, (2) an upper Pleistocene aquifer characterized by a fining upward sequence of gravel to fine sand, (3) a lower Holocene aquifer in large sand lenses embedded in floodplain deposits, and (4) an upper Holocene aquifer consisting of sandy silt (Winkel et al., 2011). Average hydraulic conductivities in the aquifers are on the order of 10^−4^ m/s (Larsen et al., 2008; Jusseret et al., 2009). The hydraulic conductivity of fine-grained floodplain sediments is on the order of 10^−8^ m/s (Jusseret et al., 2009). A downward hydraulic gradient from Holocene to Pleistocene aquifer is dominant (Larsen et al., 2008; Bui et al., 2012).

The regional groundwater flow direction is NW–SE, towards the Gulf of Tonkin, except for the depression cone formed in the Pleistocene aquifers by groundwater abstraction of Hanoi (Bui et al., 2012). The regional flow system is recharged by mountains bordering the floodplain (Larsen et al., 2008). Additionally, shallow local flow systems exist. The recharge of local flow systems occurs through floodplain sediments, also from small water bodies, and is nearly vertical with a flow rate of 0.2–1 m/a (Sø et al., 2018a). Groundwater of local systems discharges into rivers with the Red River as a main discharge zone (Larsen et al., 2008; Postma et al., 2012; Jakobsen et al., 2018). Rapid fluctuations of hydraulic heads and river stages in the monsoon season result in alternating gaining and losing periods for the Red River (Larsen et al., 2008). The reversals of the hydraulic gradient are seen up to 1.5 km from the Red River (Larsen et al., 2008).

The Holocene delta formation was initiated 9 ka BP during a post-glacial marine transgression (Tanabe et al., 2006). The sea level in the region of the Red River delta rose from −120 to 0 m in the period from the late glacial maximum to 7 ka BP. A 2–3 m sea level maximum was noted between 6 and 4 ka BP. The delta aggregation slowed down in that period and prograded again during the regression to present sea level that took place from 4 to 0 ka BP (Tanabe et al., 2006; Funabiki et al., 2012a). The Day River flowing along the SW boundary of the Red River delta was a former major channel of the Red River (Funabiki et al., 2012b).

Alluvial, tidal and marine sediments were deposited in a sedimentary basin located in a NW–SE trending fault system (Rangin et al., 1995). The left-lateral strike-slip motion of the fault system stopped at 5.5 Ma and caused an offset of a few tens of kilometers (Rangin et al., 1995). The recent sedimentation rate on the shelf of the sedimentary basin is low (0.05–0.1 mm/a) and suggests a tectonic uplift of the area (Schimanski and Stattegger, 2005). The Red River delta subsides 0.04–0.12 mm/a (Mathers and Zalasiewicz, 1999). In the central part of the basin there is an incised valley cut 30 m into the Pleistocene land surface during the last glacial maximum low sea level stand (Schimanski and Stattegger, 2005; Tanabe et al., 2006). The valley is filled with fluvial sediments followed by estuarine mud deposited during rapid sea level rise and sand and clay of alluvial environments prograding during the period of stabilized sea level (Jusseret et al., 2010; Funabiki et al., 2012a).

The thickness of the Quaternary sequence varies from a couple of meters in the proximal parts of the delta to 200 m in the coastal area (Nghi et al., 1991; Mathers and Zalasiewicz, 1999; Jusseret et al., 2010). Quaternary sediments were deposited in five cycles (Nghi et al., 1991). Lower to Upper Pleistocene gravel of a regressive system tract (cycle 1) and sea level minimum (cycle 2) are overlain by Upper Pleistocene gravel, sand and clay of a transgressive system tract deposited in alluvial, lacustrine and swamp environments (cycle 3, Nghi et al., 1991), Lower to Middle Holocene fine-grained deposits of mangrove flats and beach ridge strand plain formed during the sea level high stand 6–4 ka BP (cycle 4, Nghi et al., 1991; Tanabe et al., 2006), and Upper Holocene coarse-grained sediments of channel belts deposited mainly 3–0 ka BP (cycle 5, Nghi et al., 1991; Winkel et al., 2008). The average sedimentation rate before the sea level high stand was 8 mm/a (Funabiki et al., 2012b). Since the middle Holocene only a few meters of fine-grained floodplain sediments were deposited in the lowland areas (Funabiki et al., 2012b). Pre-Quaternary deposits comprise Precambrian bedrock, Paleozoic to Mesozoic sandstones, mudstones and limestones and Neogene clay.

## 3 Methods

### 3.1 Sedimentary structure and groundwater As at delta scale

The relationship between sedimentary structure, comprising depositional environments and shallow lithology of the Red River delta, and the groundwater As concentration was obtained from existing datasets. The architecture of the Red River floodplain to shallow depths of 10–25 m was derived from images of the Landsat Thematic Mapper integrated with compilations of shallow geological data by Mathers and Zalasiewicz (1999). The satellite images were interpreted using RGB bands 4, 5 and 7.

Winkel et al. (2011) measured the groundwater As concentration in 512 private tubewells. Each tubewell was pre-pumped for 15–30 min and groundwater samples were collected after the O_2_ level measured on-site reached a stable value. The samples for As analysis were filtered in the field through 0.45 μm cellulose nitrate filters, acidified with 1% HNO_3_, and stored in the dark at 4 °C until analysis. As concentrations were measured in triplicates with high-resolution, inductively-coupled-plasma mass spectrometry (HR ICP-MS) and cross-checked by atomic fluorescence spectroscopy (AFS). The estimated accuracy of the measurements, based on the reference samples and cross-checking, was ±5% (Berg et al., 2008; Winkel et al., 2011).

**Fig. 2:**
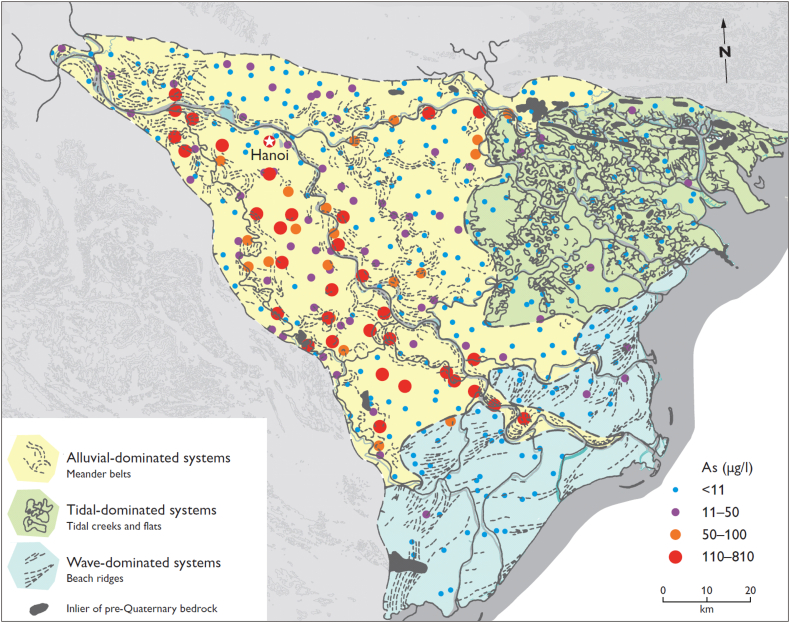
The relation between groundwater arsenic (As) content and sedimentary environment derived by remote sensing. Colour shades indicate different sedimentary environments, together with sediment structures, and were reproduced from the remote sensing work of Mathers and Zalasiewicz (1999). Colored dots indicate groundwater As concentration in household tubewells from the database of Winkel et al. (2011). (For interpretation of the references to colour in this figure legend, the reader is referred to the web version of this article.)

The map of sedimentary structure (Mathers and Zalasiewicz, 1999) was combined in ArcGIS (ESRI, 2011) with groundwater As concentrations (Winkel et al., 2011; Fig. 2). Private tubewells with As exceeding the WHO guideline of 10 μg/L were counted in each of the sedimentary environments and patterns of As distribution in the alluvial system were analyzed. A statistical analysis was performed indicating the minimum, maximum, median and 25th and 75th percentiles of groundwater As concentrations in wave-dominated, tidal-dominated and alluvial-dominated systems. Groundwater As levels in the alluvial system were analyzed in two groups, meander belts and the remaining parts of the alluvial-dominated system. Significance of the difference between means was estimated using Analysis of Variance (ANOVA).

### 3.2 Sedimentary structure and groundwater As in the alluvial environment

#### 3.2.1 Remote sensing

Fluvial structures in the proximal part of the delta, comprising courses of the paleo rivers, were traced on satellite images and a SRTM (Shuttle Radar Topography Mission) Digital Elevation Model (DEM) with the use of multiple ground reference observations and Geographic Object Based Image Analysis (GEOBIA; Blaschke et al., 2014). The applied DEM was a Digital Terrain Model (DTM) with a horizontal resolution of 30 m and vertical Root Mean Square Error (RMSE) of 16 m, although the vertical accuracy varies a lot in relation to the complexity of the terrain (e.g. steep topography). In the study area, the relative accuracy (of relevance to this study) is expected to be good due to the nature of the landscape. Ground reference observations contained information on the elevation, surface material and surface texture. Measured elevations were used to assess an uncertainty of the DEM model used in the analysis.

The GEOBIA analysis was conducted in eCognition on a Landsat 7 Thematic Mapper (USGS, 2019) scene from November 2000, recording a low flood situation (Fig. 4) and a DEM (Fig. 5). The Landsat 7 scene was recorded in eight bands and, like the DEM model, had a horizontal resolution of 30 m. A scene with the lowest level of the cloud cover and supporting the best topographic features visible in a DEM model was selected. Pixels in proximity and with similar spectral characteristics were grouped into segments that were classified into geographical features (waterbodies, bank settlements, fluvial paleostructures, roads and dikes) based on their geometry, position, texture and elevation (Fig. 4B). Pixel values in the DEM were classified into polygons representing recent Red River aggregation, highlands (elevation ≥20.2 m a.s.l.), floodplain (elevation range 10.4–20.1 m a.s.l.) and low lying floodplain (elevation ≤10.3 m a.s.l.). Additionally, analysis of the Landsat 7 satellite images from the past 50 a (1964–2015) was used to estimate the historical extent and the movement rate of the recent Red River channel belt (Fig. 4B).

#### 3.2.2 Geophysics and geology

The location and geometry of the Holocene river channels and floodplains, Pleistocene sand and clay units and bedrock elevation were delineated along five cross-sections based on gamma logging (Figs. 1B and 6) and ~ 50 km of resistivity profiles measured by Continuous Vertical Electrical Soundings (CVES, Figs. 1B and 7). Five major resistivity cross-sections were measured in sub-sections of 410–890 m using SYSCAL PRO equipment. The sub-sections of 410–890 m were overlapped by 100 m to cross-check the results and ensure accuracy and repeatability of the resistivity measurements. Each of the major resistivity cross-sections was placed in the vicinity of a few boreholes with a well-known geology (Fig. 1B). The Wenner method with 96 electrodes positioned with 5 m spacing giving a penetration depth of up to 70 m was used. The resistivity profiles were processed in Aarhus Workbench using laterally constrained inversion (Auken et al., 2009). The noise levels were estimated by comparing the original and corresponding reciprocal measurements and the RMSE was estimated for each inverse resistivity model. Measured resistivity profiles were interpreted using geological borehole information, gamma log profiles and resistivity ranges in the Red River delta described by Tran et al. (2012). The resistivity profiles were divided into five major lithological units: (1) Holocene sand of the channel belts, (2) Holocene silt and clay deposited on the floodplain and in abandoned oxbow lakes, (3) Pleistocene clay, (4) Pleistocene sand and gravel and (5) bedrock.

Geological borehole information was compiled from the database of National Center for Water Resources Planning and Investigation (57 boreholes), 5 boreholes described in Larsen et al. (2008) and Postma et al. (2012), and 14 boreholes drilled during the present study. The boreholes were drilled using jet drilling to depths of 13–42 m below the terrain surface. Water used during the drilling originated from nearby boreholes. The geological information was derived from cuttings collected at the surface. Natural gamma radiation was measured in 19 boreholes using Robertson Research Ltd. equipment (Keys, 1990) to cross-check the geological borehole information.

#### 3.2.3 Sediment age

The burial age of the sandy deposits was measured with Optically Stimulated Luminescence (OSL) at the Nordic Laboratory for Luminescence Dating, Denmark (Murray and Wintle, 2000, Murray and Wintle, 2003). 18 sediment cores were taken using 3 m long stainless steel tubes on a barrel-free piston corer (Starr and Ingleton, 1992) and stored in segments of the stainless steel tubes preventing light exposure. The cores were stored frozen until analysis (Sø et al., 2018a). From each core a set of sub-cores for the OSL dating was selected based on differences in natural gamma radiation and lithology. In total, 39 sub-cores, each 20 cm in length, were cut out under red light from the main cores from depths of 6.6–38 m below terrain surface and sealed to prevent light exposure prior to the burial age measurement. Quartz grains were extracted at the laboratory and used for dating (Murray and Wintle, 2000, Murray and Wintle, 2003). The accuracy of the sediment burial age is ±100 a (Sø et al., 2018a). The adopted analysis protocol is described in detail in Sø et al. (2018a).

In addition, relative sediment age was interpreted using archeological data from the Red River delta. The location and age of Holocene settlements (Funabiki et al., 2012b) were compiled in ArcGIS (ESRI, 2011) together with the Digital Elevation Model (Fig. 5). All settlements were found at depths of a few meters below terrain surface and indicated the relative age of under- and over-lying deposits.

#### 3.2.4 Geological modeling

The 3D sedimentology and stratigraphy of the Red River floodplain was interpreted using remote sensing, geophysical, geological, archeological and OSL data. The datasets were combined in GeoScene 3D. The interpreted lithological units were gravel, sand and clay/silt and divided further into 16 stratigraphical units (Fig. 8). The lithological units in boreholes and gamma log profiles were interpolated using the remote sensing information and geophysical data as interpolation constraints. The resistivity profiles and the extent of channel belt and floodplain deposits interpreted from remote sensing data, indicated an approximate border between different lithologies where no borehole data existed. Archeological and OSL data were used to assign relative or specific ages to the lithological units. Interpreted sedimentological and stratigraphical layers were interpolated in ArcGIS (ESRI, 2011) and visualized in Groundwater Modeling System (GMS).

#### 3.2.5 Groundwater As concentration

Groundwater As concentrations in the alluvial environment were compiled from previous studies (Berg et al., 2008; Winkel et al., 2011; Sø et al., 2018a) and supplemented by analyzing an additional 48 private tubewells and two piezometer fields. Detailed groundwater sampling and analysis procedures are described in Sø et al. (2018a). Each piezometer was equipped with a 1 m long screen, pumped after the completion of drilling to remove any water used during the drilling and left to equilibrate for at least three months prior to groundwater sampling (Sø et al., 2018a). Five piezometer or tubewell volumes were flushed with a submersible pump before the groundwater sample was taken. O_2_, EC and pH were measured on-site in a flow cell. Groundwater samples were filtered through 0.2 μm cellulose acetate filters. The samples for As analysis were acidified with 2% HNO_3_ and refrigerated. Arsenic was analyzed by atomic absorption spectrophotometry, using a HVG hydride generator and a graphite furnace at the Research Centre for Environmental Technology and Sustainable Development (CETASD), Hanoi University of Science. The device was calibrated against certified reference samples. The detection limit for As was 0.013 μM (Sø et al., 2018a). In the Appendix of Winkel et al. (2011) an extensive quality assurance and control is presented, using certified international reference samples and cross-evaluation between different analytical techniques applied in the CETASD laboratory in Vietnam and by Winkel et al. (2011) at EAWAG in Switzerland, e.g. AAS versus ICP-MS and AFS. The results of certified samples and cross-checking between methods agreed within ±5%. Berg et al. (2008) applied the same sampling and analysis procedures as Winkel et al. (2011).

The groundwater As concentration in 94 samples was compared with the interpreted architecture of the alluvial system. A statistical analysis was performed indicating the minimum, maximum, median and 25th and 75th percentiles of groundwater As concentrations in four areas of shallow sedimentary and stratigraphy structure. The considered areas were: (1) Pleistocene clay terraces and sand islands, (2) thick Early to Mid-Holocene floodplain deposits underlain by Pleistocene and Holocene sand, (3) old channel belt deposits buried 5.9–2.9 ka BP, and (4) younger channel belt deposits buried 1.7–0.4 ka BP. The significance of the difference between means was estimated using ANOVA.

## 4 Results

### 4.1 Delta scale sedimentology, stratigraphy and As distribution

Delta deposits consist of sediments with a large spatial variation in sediment type and age. Remote sensing (images of the Landsat Thematic Mapper) was used by Mathers and Zalasiewicz (1999) to analyze the sedimentary architecture of the Red River delta plain. Their results are reproduced in Fig. 2 and show three distinct sedimentary environments. The NE part of the delta is dominated by tidal-dominated systems, while in the SE part wave reworked shoreface sediments are found. The area further away from the coast consists of alluvial-dominated sediments comprising meander belts, anastomosing rivers, floodplains and fluvial terraces. The meander belt deposits identified by remote sensing are also shown on the map (Fig. 2).

**Fig. 3:**
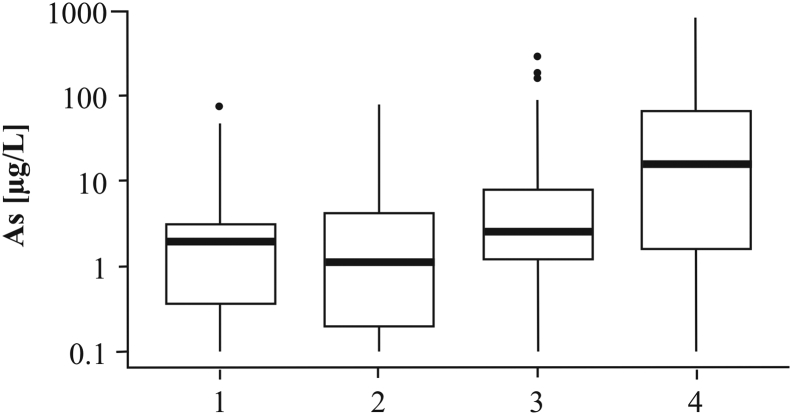
Arsenic box plot in the Red River delta. The median (thick black line), 25th and 75th percentiles (bottom and top edge of the box, respectively), most extreme observations excluding outliers (whiskers) and outliers (dots) were plotted for: (1) wave-dominated system, (2) tidal-dominated system, and alluvial-dominated system divided into (3) undisturbed floodplain and (4) meander belts.

Winkel et al. (2011) published a detailed map of the As distribution in groundwater of the Red River delta plain, based on >500 samples of household tubewells distributed throughout the entire delta plain. Arsenic concentrations ranged from <0.1 to 810 μg/L. These results were superimposed on the map of sedimentary environments of Mathers and Zalasiewicz (1999). The results (Fig. 2, Fig. 3) show a predominant occurrence of high As groundwater in the SW alluvial part of the delta plain comprising floodplain and meander belts, but also display a very high variability in groundwater As content.

In the wave-dominated system there are only three boreholes with 11–50 μg/L As, i.e. above the WHO guideline of 10 μg/L As, and one with 50–100 μg/L As at the boundary to the alluvial-system area. The remaining 58 boreholes in the wave-dominated area all have <11 μg/L As (Fig. 2). The median groundwater As concentration is 1.9 μg/L, 25th percentile equals to 0.4 μg/L As and 75th percentile to 3 μg/L As (Fig. 3). In the tidal-dominated zone, there are six boreholes with 11–50 μg/L As while the remaining 70 boreholes have <11 μg/L As (Fig. 2). The median groundwater As concentration in the tidal-dominated zone is 1.1 μg/L, 25th percentile equals to 0.2 μg/L As and 75th percentile to 4.2 μg/L As (Fig. 3). Of the 244 analyzed tubewells in the alluvial part of the delta, 30% contain more As than the WHO guideline of 10 μg/L As and in 20% of the tubewells the As content is greater than 50 μg/L. Within the alluvial sediments the distribution of observed meander belts is indicated in Fig. 2 and it shows a good correspondence with the occurrence of high As groundwater. Out of the 33 tubewells containing more than 110 μg/L As, only three boreholes are not associated with meander belt observations. Median groundwater As concentrations in the meander belts and the remaining areas of the alluvial system are 16 μg/L and 2.5 μg/L, respectively. The 25th percentile is similar in both environments – 1.6 μg/L As in the meander belts and 1.2 μg/L As in the remaining parts of the alluvial system. Groundwater As concentrations vary greatly for the 75th percentile and are 66 μg/L As in the meander belts and 8.1 μg/L As in the remaining parts of the alluvial system (Fig. 3). The statistical analysis indicates that low As levels are connected to the marine deposition environments (wave-dominated and tidal-dominated), and the highest As levels are found in the meander belts (Fig. 3). ANOVA indicated a significant difference between the groups with a *P*-value of 1 × 10^−10^.

### 4.2 Sedimentology and stratigraphy of the alluvial system

#### 4.2.1 Remote sensing

**Fig. 4:**
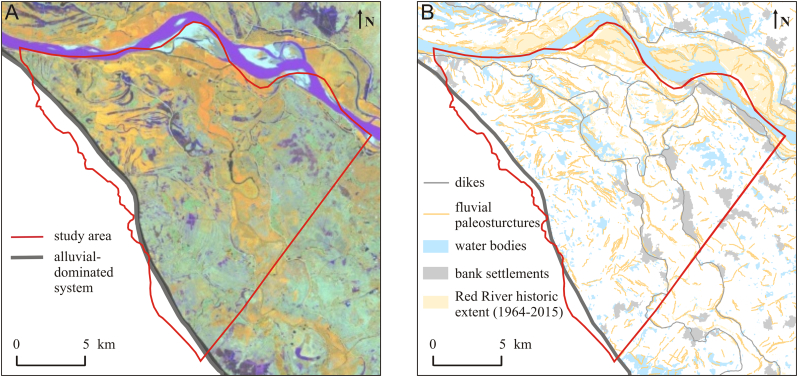
(A) Subset of a LANDSAT 7 scene covering the study area (colour composite image is shown as RGB; bands 3, 4, and 5). Dark blue curves indicate the location of old river meanders. (B) Distribution of fluvial paleostructures, bank settlements, water bodies and Red River historic extent classified from remote sensing data 1964–2015. Densely distributed sinusoidal fluvial paleostructures indicate the location of old sandy channel belts, in which, during the wet season, a formation of elongated water bodies is often observed. Villages with elongated shape as a result of placement of settlement at elevated river banks (bank settlements) are an additional indicator of previous river courses. Construction of dikes in the Red River started at 1 ka BP and greatly limited river migration. (For interpretation of the references to colour in this figure legend, the reader is referred to the web version of this article.)

Fluvial paleostructures in the Landsat 7 scene used for the classification of geomorphological features (GEOBIA) are visible as (1) elongated, curved, thin features in the landscape representing abandoned meander belts, (2) residual water bodies, and (3) villages with an elongated, curved shape (bank settlements), Fig. 4. The bank settlements are often placed on the higher elevated paleo levees along the river banks (Fig. 4; Weinman et al., 2008). The water bodies in the research area are represented by oxbow lakes and crevasse splays. Oxbow lakes are depositional remnants of the abandoned channel belts, and crevasse splays represent areas of floodplain sedimentation.

**Fig. 5:**
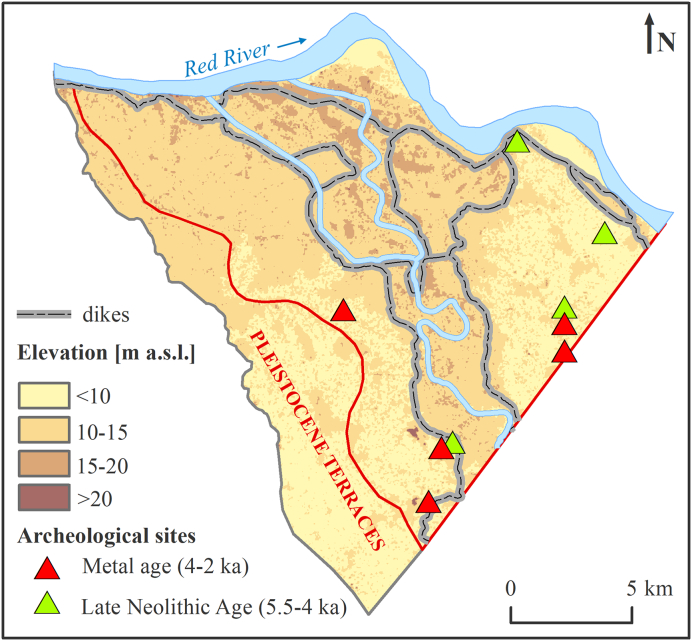
Age of near-surface sediments interpreted from archeological data and a digital elevation model. High-lying (>10.3 m a.s.l.) floodplain bordered by dikes was deposited in the past 1 ka, while low-lying areas (≤10.3 m a.s.l.) consist mostly of sediments older than 2 ka, based on the age of archeological sites excavated 1 m below the terrain surface. Settlements of the late Neolithic Age scattered in the NE part of the research area indicate that major alluvial deposition at these sites ended before 5.5 ka BP.

Groups of sinusoidal paleo river scars indicate the locations of old channel belts and are most abundant along the SW boundary of the research area and along the recent Red River course (Fig. 4B). The bank settlements were built adjacent to the old channel belts, following their shape. The higher elevated areas are most abundant along the recent Red River and Day River courses (Fig. 5) and represent point bar and levee deposits. Higher elevations of these areas are also an effect of a constrain on recent alluvial deposition within the dikes. The movement of the active river channel occurs within a 1-km-wide zone and the deposition of a 500-m-wide point bar island currently happens on a time scale of a couple of years (Fig. 4B). A similar distribution of the paleo rivers is visible in the Landsat 7 image (colour composite image is shown as RGB; bands 3, 4, and 5), with abandoned meander belts standing out as dark blue curves (Fig. 4A). Scarce fluvial paleostructures, irregular shape of the water bodies (Fig. 4) and low, uniform terrain elevation (Fig. 5) in the NE corner of the research area, all indicate floodplain rather than channel belt deposition.

#### 4.2.2 Geophysics

**Fig. 6:**
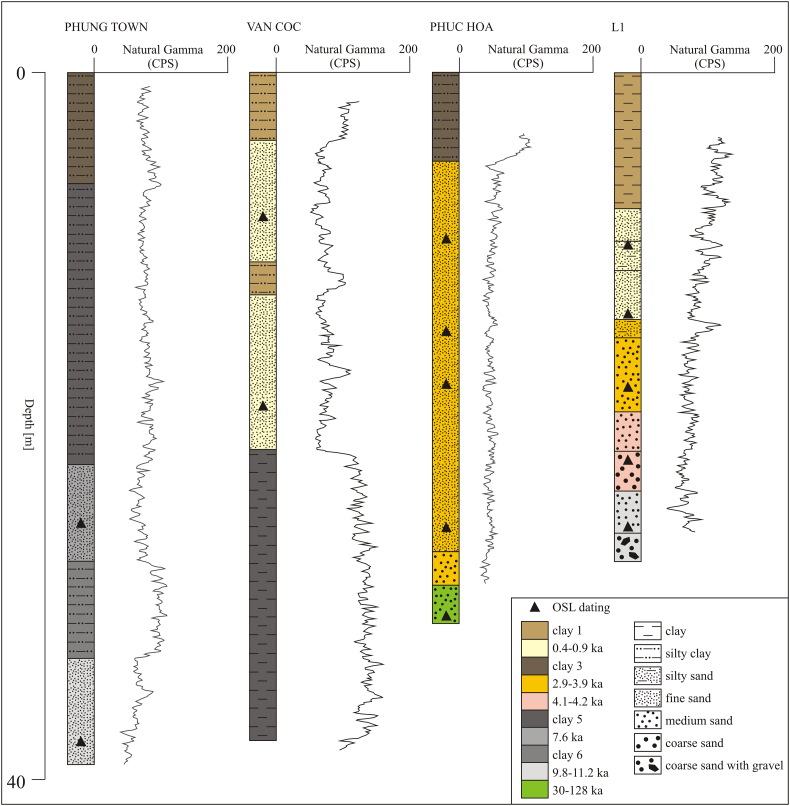
Sediment sequences in the Red River delta observed directly during borehole installation (lithological profiles) and in natural gamma-logging compared with results of Optical Sediment Luminescence (OSL) dating. Location of boreholes is marked in Fig. 1B. (For interpretation of the references to colour in this figure legend, the reader is referred to the web version of this article.)

Fining-upwards sequences of the alluvial deposition are marked as upwards increasing natural gamma radiation values in the gamma log profiles (Fig. 6). Up to 20-m-thick sand sequences in the channel belts outside of the dikes have uniform natural gamma radiation values (average 68 CPS) and are covered by thin layers (up to 5 m) of fine-grained floodplain deposits with an increasing natural gamma radiation averaging 102 CPS (e.g. Phuc Hoa profile in Fig. 6). Silt and clay deposited in mangrove and floodplain environments in the lowland areas have a natural gamma radiation in the range of 61–108 CPS (Phung Town profile in Fig. 6). Here, an up to 30 m thick fine-grained sequence is underlain by sand with natural gamma radiation averaging 64 CPS. Sand of recent channel belts of the Day River and Red River has an average gamma radiation of 75 CPS, and clay and silt on average 106 CPS. Deposits along the Day River, inside of the dikes, comprise sand of multiple channel belt sedimentation cycles covered by fine-grained floodplain deposits (L1 profile in Fig. 6). These sedimentation cycles produced a gamma radiation profile consisting of multiple sub-profiles with overall upwards increasing CPS values. A similar trend occurs along the recent Red River channel where alternating layers of sand and clay were observed (Van Coc profile in Fig. 6). There is a clear gamma-radiation boundary between coarse-grained channel belt deposits (avg. 72 CPS) and fine-grained floodplain sediments (avg. 132 CPS) underlying the recent Red River sediments.

**Fig. 7:**
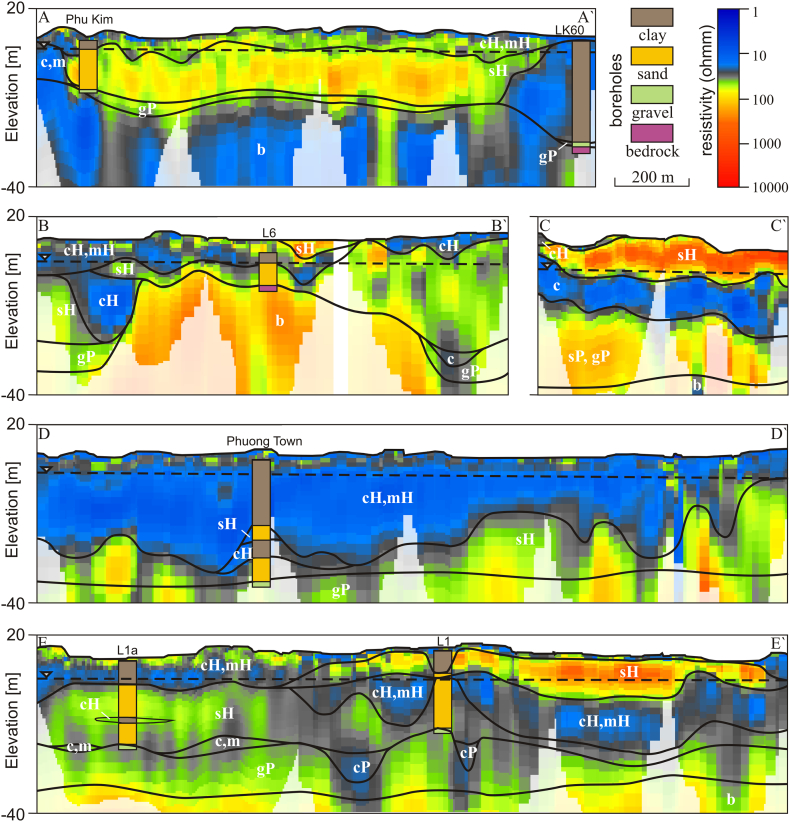
Distribution of coarse-grained channel deposits and fine-grained deposits of the floodplain based on CVES data. Lithological symbols are: c (clay), m (silt), s (sand), g (gravel), H (Holocene) and P (Pleistocene). (A) Areas outside of dikes. Mid-Holocene channel belts intersect thick fine-grained floodplain deposits. (B) Areas inside of dikes along the Day River. Recent and mid-Holocene channel belts deposited around outcropping bedrock. (C) Areas close to the recent Red River course. Mid-Holocene deposits are eroded and replaced by recent channel belt sediments. (D) Areas outside of dikes. Fine-grained floodplain deposits. (E) Areas inside of dikes along the Day River. Recent channel belts (<1 ka BP) cut through the uppermost part of the mid-Holocene sequence. (For interpretation of the references to colour in this figure legend, the reader is referred to the web version of this article.)

Results of the CVES survey (Figs. 1B and 7) interpreted based on geological borehole information (Fig. 6, Fig. 7), gamma-logging (Fig. 6), and results of the study by Tran et al. (2012) show great spatial variability. Electrical resistivity of fine-grained floodplain deposits (clay, silt) ranges 10–50 ohmm and is mainly up to 30 ohmm. The resistivity of sand and gravel in the channel belts ranges from 50 ohmm in the saturated zone to over 1000 ohmm in the unsaturated zone (Fig. 7). Sediments outside of the dikes are either dominated by the sandy deposits of the channel belts (Fig. 7A) or by fine-grained floodplain deposits (Fig. 7D). The spatial extension of the latter corresponds to the NE lowland areas in the landscape (Figs. 1B and 5). The fine-grained floodplain deposits have a thickness of up to approximately 34 m, contain thin Holocene sand lenses and are underlain by Holocene and Pleistocene sand and gravel (Fig. 7D). Old channel belts filled with sand are concentrated in the SW area (Fig. 1B). They display a fining-upwards sequence with sand layers up to approximately 38 m thick covered by a thin (<10 m) clay layer (Fig. 7A). Areas inside of the dikes comprise sandy deposits of the old channel belts and floodplains partially eroded by the Day River and Red River and filled up with recent fining-upwards sand-clay sequences (Fig. 7B, C and E). Locally, a Quaternary sequence is deposited around inselbergs of the pre-Quaternary sandstones and limestones characterized by high resistivity values, >100 ohmm (Fig. 7B). RMSE of the inverse models of the sub-section resistivity profiles ranged from 0.5–5% with an average of 1.9%.

#### 4.2.3 Sediment age

The age of the settlements discovered in upper layers of the Red River floodplain varies from 5.5 ka (Late Neolithic Age) to 2 ka (Metal Age), Fig. 5 (Funabiki et al., 2012b). The oldest settlements were found in the lowlands of the north-eastern and southern part of the research area and along the dikes (Fig. 5). Radiocarbon dating was done in a 16.6 m deep borehole located close to the southern lowlands, in a meandering belt of the Day River by Funabiki et al., 2012a. The calibrated age of wood and organic material ranged from 5.1 to 8.3 ka (Funabiki et al., 2012a).

**Fig. 8:**
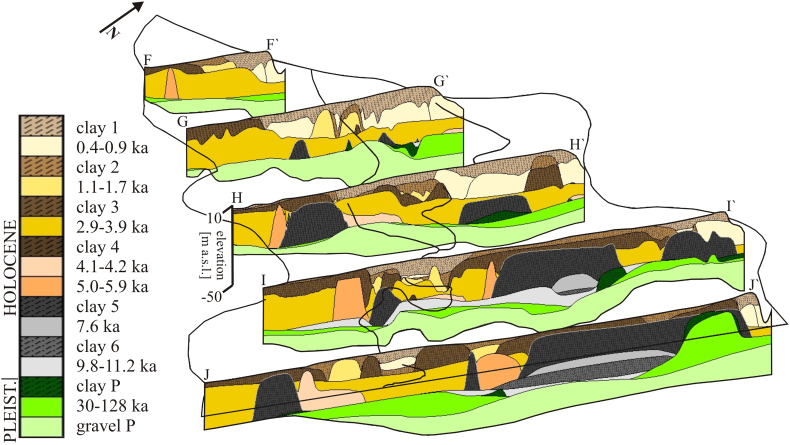
Fence diagram of the compiled geological model of the Red River floodplain within the surveyed area in the uppermost part of the Red River delta. (For interpretation of the references to colour in this figure legend, the reader is referred to the web version of this article.)

**Fig. 9:**
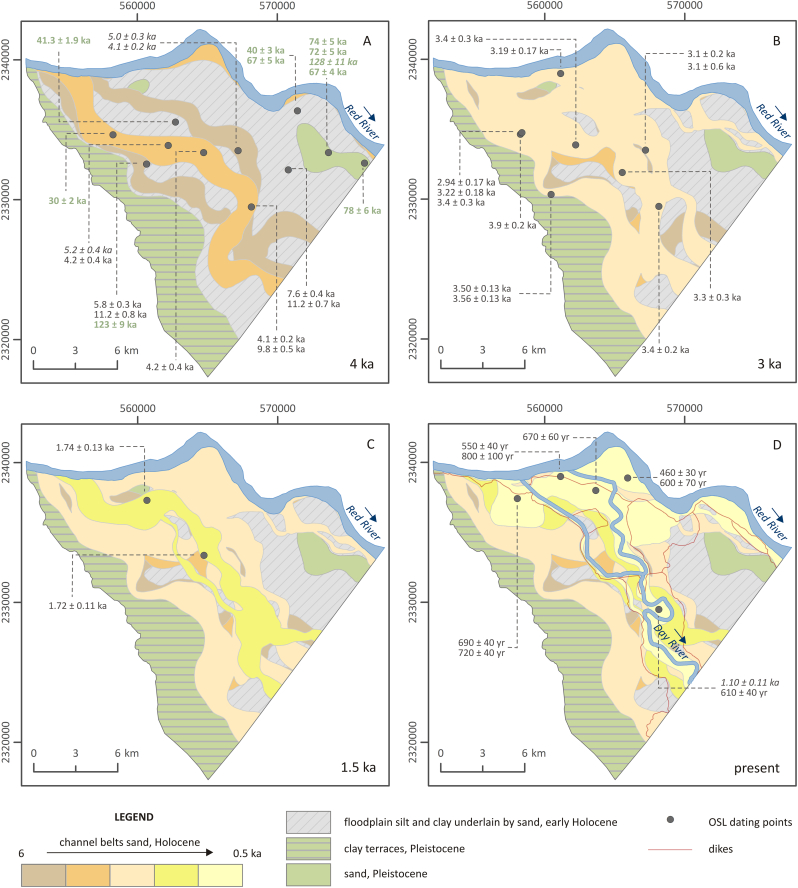
Geological evolution of the uppermost part of the Red River delta interpreted based on remote sensing, geophysical, geological and archeological data calibrated against OSL dating. OSL ages for the Pleistocene sediments are in green colour. (For interpretation of the references to colour in this figure legend, the reader is referred to the web version of this article.)

The burial age of sand on the Red River floodplain, measured by OSL, ranges from 128 ± 11 ka to 460 ± 30 a (Fig. 6, Fig. 8, Fig. 9). The horizontal Pleistocene layers underlying Holocene sediments were deposited between 128 ± 11 ka to 30 ± 2 ka (Fig. 9A). The majority of Holocene channel belts outside of the dikes contain sand dated from 3.9 ± 0.2 ka to 2.94 ± 0.17 ka (Figs. 6 and 9B). The oldest channel belt deposits are dated to 5.8 ± 0.3 ka (Fig. 8, Fig. 9A). Late Holocene sand deposited inside of the dikes has an age of 1.74 ± 0.13 ka to 460 ± 30 a (Figs. 6, 9C and D). Fine-grained mangrove deposits of the lowland area are underlain by Holocene sand dated to 11.2 ± 0.7 ka to 7.6 ± 0.4 ka (Figs. 6 and 9A).

#### 4.2.4 Geological evolution of the alluvial system

Detailed stratigraphy and sedimentology of the alluvial system are shown in Fig. 8. Pleistocene gravel and sand layers with a thickness of up to approximately 48 m are present throughout the entire area and overlain by either a Pleistocene clay layer or various Holocene sediments. Early Holocene sand and fine-grained floodplain and river mouth deposits are preserved in the NE part of the research area and were deposited around an island of Pleistocene sand. In the SW part of the floodplain early Holocene deposits and sand of older channel belts occur only locally and are replaced with sand dated to 2.9–3.9 ka BP. Inside of the dikes, sediments are dominated by late Holocene sand and clay (<1 ka BP).

Fig. 9 shows the Holocene evolution of the Red River floodplain interpreted based on data described in sections 4.2.1, 4.2.2 and 4.2.3. The oldest channel belt sand is dated to 5.8 ± 0.3 ka BP (Fig. 9A), which corresponds to alluvial deposition in the period of the Holocene sea level maximum (Tanabe et al., 2006). Before the construction of dikes, which began at approximately 1 ka BP, the Red River mainly moved throughout the SW part of the floodplain while fine-grained floodplain deposits and Pleistocene sand in the NE area remained undisturbed (Fig. 9A, B and C). Sedimentation of the recent deposits takes place mainly inside of the dikes, where older channel belt and floodplain deposits are eroded and replaced by younger sediments (Fig. 9D).

### 4.3 Groundwater As distribution in the alluvial system

**Fig. 10:**
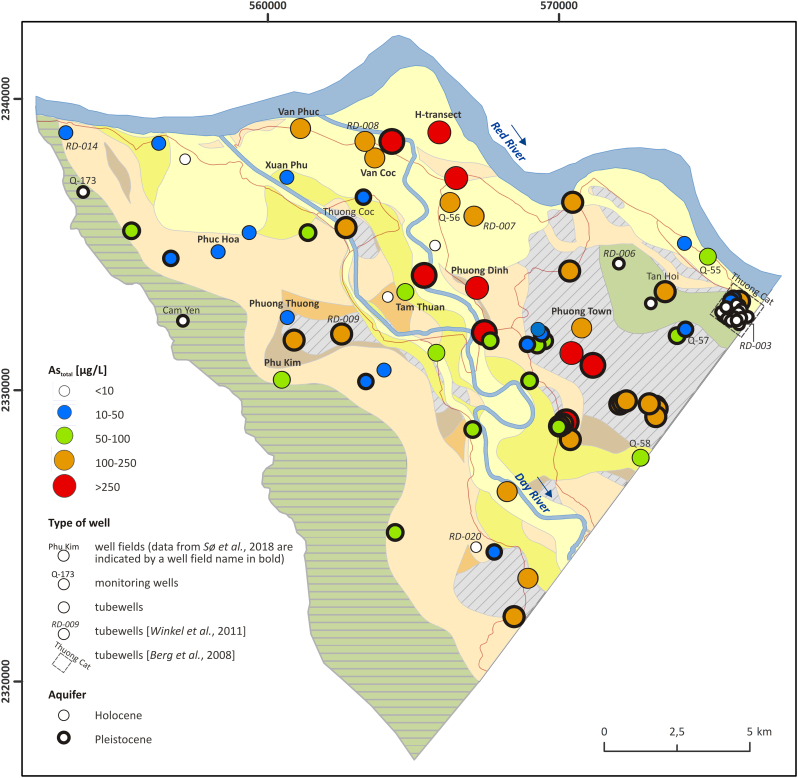
Relation between groundwater As content and geological features and evolution of the uppermost part of the Red River delta. Description of the geological units is given in Fig. 9. (For interpretation of the references to colour in this figure legend, the reader is referred to the web version of this article.)

**Fig. 11:**
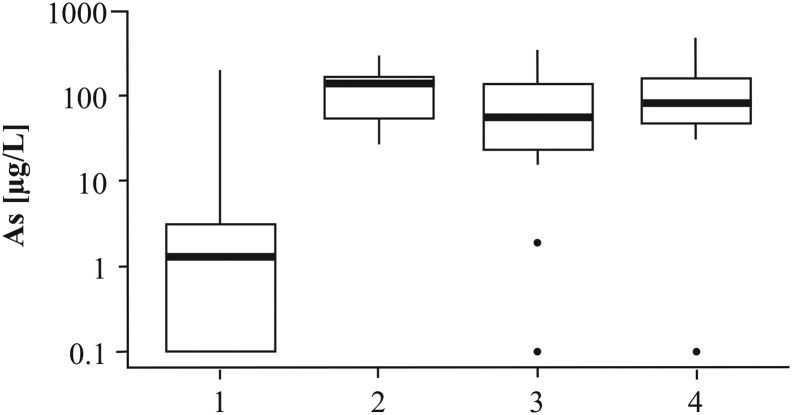
Arsenic box plot in the proximal, alluvial part of the Red River delta. The median (thick black line), 25th and 75th percentile (bottom and top edge of the box, respectively), most extreme observations excluding outliers (whiskers) and outliers (dots) were plotted for: (1) Pleistocene clay terraces and Pleistocene sand islands, (2) areas with thick, undisturbed fine-grained floodplain deposits, and meander belts divided into two age groups: (3) 2.9–5.9 ka, and (4) 0.4–1.7 ka.

Groundwater As concentrations in the studied part of the alluvial system range from 0 to 488 μg/L (Fig. 10, Fig. 11). The highest median As concentrations were found inside of dikes that constrain the channel belts with a sediment burial age < 1.7 ka (85 μg/L As) and below the thick, undisturbed fine-grained floodplain deposits (143 μg/L As), Fig. 11. All boreholes with a screen below the thick, fine-grained floodplain deposits exceed the WHO guideline of 10 μg/L As. In 32% of wells, As concentrations are higher than 100 μg/L including wells screened in the Pleistocene aquifer (Fig. 10). The 75th percentile As concentration equals 169 μg/L (Fig. 11). Out of 27 wells in the area of channel belts filled with sand of an age < 1.7 ka, only 2 boreholes have <10 μg/L As and 48% of the wells have >100 μg/L As. In the older meander belts buried 5.9–2.9 ka BP, groundwater As concentrations are lower with a median As level of 56 μg/L (Fig. 11). Out of 19 boreholes, 2 have <10 μg/L As and only 26% of the wells >100 μg/L As (Fig. 10). The lowest As concentrations occur in groundwater sampled below Pleistocene clay terraces and in the Pleistocene sand island near Thuong Cat (Fig. 10). The median groundwater As level in these areas is 1.2 μg/L (Fig. 11) and only 4 out of 52 boreholes have As groundwater concentrations >10 μg/L. ANOVA indicated a significant difference between the groups with a *P*-value of 5 × 10^−5^.

## 5 Discussion

### 5.1 Groundwater As vs. deposition environment at the delta scale

A strong relationship between the sedimentary environment derived from remote sensing (Mathers and Zalasiewicz, 1999) and the groundwater As content (Winkel et al., 2011) of the Red River delta is revealed in Fig. 2, Fig. 3 with the predominant occurrence of high As groundwaters in the alluvial dominated system, in the SW part of the delta plain, though showing a high variability in the groundwater As content. The marine influenced sedimentary environments, the tidal-dominated and the wave-dominated coastal deposits (Fig. 2), have a low groundwater As content (Fig. 3). Marine sediments initially contain seawater rich in dissolved sulfate. Under anoxic conditions, organic carbon reduces sulfate to dissolved sulfide, which leads to precipitation of As in sulfide mineral phases (Quicksall et al., 2008; Buschmann and Berg, 2009). Presumably this is the main reason for why the marine sediments of the Red River delta contain groundwater low in As (Jessen et al., 2008).

The high As groundwater tubewells (>100 μg/L As) are all found in the alluvial part of the delta (Fig. 2, Fig. 3) and are mainly associated with the meander belts (Fig. 2, Fig. 3, Fig. 10) and thick undisturbed fine-grained floodplain sediments (Fig. 10, Fig. 11). Groundwater in the part of the alluvial-dominated system without pronounced alluvial features has lower groundwater As levels (Fig. 2, Fig. 3). Abandoned meander belts and thick floodplain deposits contrary to the older terraces may be a source of fresh organic matter for iron oxyhydroxides reduction and associated As release (Kocar et al., 2014; Postma et al., 2016). Furthermore, thick clay layers limit the extent of As flushing from the aquifer (Sø et al., 2018a). These relationships are further explained in the paragraph below and section 5.2. The distribution of meander belts indicates the presence of an abandoned river channel along the SW margin of the floodplain (Tanabe et al., 2006; Jessen et al., 2008) which corresponds to high As groundwater (Fig. 2, Fig. 3). There is also an east going abandoned river channel near the NE delta boundary, in the alluvial-dominated system east of Hanoi, which likewise shows elevated As concentrations in groundwater (Fig. 2). Finally, where the present day main channel of the Red River cuts through the wave reworked deposits, high (>50 μg/L) groundwater As concentrations are also found (Fig. 2). Similarly, Polya et al. (2005) and Papacostas et al. (2008) report that high As wells often are found close to river channels although Papacostas et al. (2008) could not determine a significant statistical difference in groundwater As concentration between wells located within one kilometer from modern river channels and those located further away.

In freshwater, the reduction of iron oxyhydroxides with associated As only leads to limited subsequent immobilization of the released As through adsorption or reprecipitation (Postma et al., 2016). From a hydrogeological and hydrochemical point of view, it is found that in alluvial sediments, the important factors that influence groundwater As concentration comprise the extent of flushing (Sø et al., 2018a; Jakobsen et al., 2018) and the availability and reactivity of organic matter influencing the reduction rate of iron oxyhydroxides and As release (Postma et al., 2012, Postma et al., 2016). These two factors are partially related to the burial age of sediments due to decreased reactivity of the organic matter (Postma et al., 2012, Postma et al., 2016) and the increasing number of pore volumes flushed over time (Sø et al., 2018a). The extent of flushing is also dependent on lithology, and thus hydrogeological properties of the sediments, groundwater recharge rates and flow patterns (Jakobsen et al., 2018; Sø et al., 2018a). Furthermore, freshly deposited floodplain sediments can be an additional source of organic matter available for iron oxyhydroxide reduction (e.g. Kocar et al., 2014). As a result, variability in the As concentration in the alluvial-dominated systems is a function of sedimentary history (Fig. 8, Fig. 9, Fig. 10) and hydrogeological regime. This is the case for the vertical sedimentary sequence but also laterally through sideward migrating meandering channels, and at a more detailed scale on the accumulating side of river meanders where groundwater may contain high As (Jakobsen et al., 2018).

At the delta scale, a few samples in marine environments have groundwater As levels >50 μg/L and some samples in meander belts have <10 μg/L As (Fig. 2, Fig. 3). Thus, all local variations in groundwater As levels cannot be captured by remote sensing at the delta scale. Furthermore, an alluvial-dominated system situated at the boundary of a marine-dominated system may be influenced by saltwater intrusion and salty paleowater found in some boreholes up to 75 km inland (Tran et al., 2012). This is confirmed by EC values >1000 μS/cm in 27% of private tubewells in the alluvial-dominated system (Winkel et al., 2011). Groundwater As levels <10 μg/L occur in 51% of private tubewells with EC >1000 μS/cm in the alluvial-dominated system and can be related to chemical processes similar to those in the marine sedimentary environments. Nevertheless, the joint interpretation of satellite images with available groundwater As measurements is useful in delineating areas with a good chance of finding groundwater low in As. The median and 75th percentile As concentrations in the alluvial-dominated system outside of the meander belts, as well as the wave- and tidal-dominated systems were all below the WHO guideline of 10 μg/L As (Fig. 3).

### 5.2 Stratigraphy, sedimentology and hydrogeology of the alluvial system vs. the groundwater As distribution

**Fig. 12:**
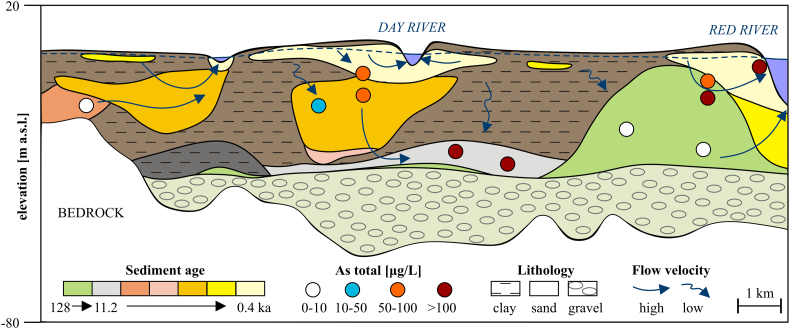
A conceptual model of the influence of sedimentology, stratigraphy and hydrogeological processes on the distribution of groundwater As in the Red River floodplain. Measured As concentrations in groundwater are influenced by transport of As contaminated groundwater from younger to older aquifers as well as by the number of pore volumes flushed. (For interpretation of the references to colour in this figure legend, the reader is referred to the web version of this article.)

The groundwater As distribution is influenced by the stratigraphy and sedimentology of the alluvial system (Fig. 9, Fig. 10, Fig. 11, Fig. 12), the resulting flow paths (Fig. 12; Kocar et al., 2014; Hoque et al., 2017), and especially near rivers by water level changes driven by dry and monsoon seasons (Larsen et al., 2008). The lowest groundwater As levels are found in the Pleistocene and early Holocene sands and gravels overlain by a thin clay layer (Fig. 10, Fig. 11). It is a result of many pore volumes flushed through these deposits due to high porewater velocities and longer exposure to recharge as compared to the fine-grained and/or younger deposits (Jakobsen et al., 2018; Sø et al., 2018a). The number of flushed pore volumes was estimated based on the vertical flux rate estimated from groundwater age, depth of an aquifer and sediment age (Sø et al., 2018a). Sø et al. (2018a) found that after about 200 pore volumes had recharged through the aquifer, the groundwater As level became <10 μg/L in agreement with the geochemical model of Postma et al. (2016). With an increase in thickness of the uppermost clay layer, the number of recharged aquifer volumes decreases, and the groundwater As concentrations stay higher even in the early Holocene deposits (Sø et al., 2018a; Fig. 10, Fig. 11, Fig. 12). The highest As concentrations are found in late Holocene aquifers due to the availability and reactivity of organic matter (Postma et al., 2012, Postma et al., 2016). In addition, As may be transported into aquifers in the older deposits where hydraulic windows occur (Fig. 12). The geochemical model of Postma et al. (2016) for the evolution of the groundwater As level in Red River aquifers over the last 6 ka, was applied in a 2D reactive transport model by Jakobsen et al. (2018), to successfully predict the observed groundwater As distribution in various settings in: Bangladesh (Weinman et al., 2008), the Mekong floodplain in Vietnam (Sato and Shibasaki, 2013) and in Cambodia (Benner et al., 2008).

The probable spatial distribution of shallow (depth 10–25 m) geological structures at the regional scale (Fig. 8, Fig. 9) was derived from remote sensing based classification of geomorphology combined with scarce shallow borehole information. Remote sensing is especially effective in delineating areas with Holocene channel belt migration (Papacostas et al., 2008; Sahu and Saha, 2015; Das et al., 2021), and thus in predicting areas with potentially high As groundwater (Fig. 10) due to the abundance of fresh organic matter. The most prominent features in the Landsat 7 scene are the geometry, location and emergence order of meander cut offs (Fig. 4). The combination of available data on groundwater As levels with areal extension of meander cut offs of different orders may help to predict groundwater As level in areas where hydrochemical data is not available (Fig. 10, Fig. 11).

A classification of channel belts by their relative age allows to identify areas of potentially high and low groundwater As (Fig. 10) as it is related to the availability and reactivity of sedimentary organic matter, decreasing with sediment age (Postma et al., 2012). The As concentration will also decrease with sediment age because more pore volumes have flushed through, removing adsorbed As, if the hydrogeological properties of the channel belts are similar (Sø et al., 2018a). This explains why less As contaminated aquifers, with a sediment burial age > 2.9 ka BP, are found along the SW margin of the alluvial system and more As contaminated aquifers are found along the recent (<1.7 ka BP) Red River and Day River channels (Fig. 10, Fig. 11). Unless covered by a thick clay layer (e.g. sand dated to 11.2 ka BP in Fig. 12), aquifers with a sediment burial age > 2.9 ka BP have had more than 100 pore volumes recharged through the system and are therefore depleted in groundwater As (Sø et al., 2018a). Aquifers in Holocene deposits with a burial age < 1 ka BP have had less than 100 pore volumes recharged and therefore contain high groundwater As concentrations (Sø et al., 2018a).

Remote sensing was also useful for delineating low lying, undisturbed floodplains probably containing Pleistocene and early Holocene aquifers, based on the lack of visible meander features in satellite images (Fig. 2, Fig. 4, Fig. 9D). Undisturbed floodplains hosting old aquifers should have a low groundwater As concentration (Papacostas et al., 2008). Low groundwater As levels were related to undisturbed floodplains in the delta scale analysis (group 3 in Fig. 3). However, in the more detailed regional analysis, high As concentrations were found in some parts of the undisturbed floodplain (Fig. 10, Fig. 11) due to the supply of organic matter from a thick overlying clay layer (Nath et al., 2010; Sahu and Saha, 2015) which also limits infiltration and flushing of the aquifer (Sø et al., 2018a). Despite the high sediment burial age > 7.6 ka BP, the number of pore volumes flushed through an aquifer covered by a > 10 m thick clay layer was only approximately 100 and resulted in >100 μg/L As in the groundwater (Sø et al., 2018a). Aquifers below Pleistocene clay terraces and in Pleistocene sand islands in the proximal undisturbed part of the floodplain have low As groundwater (Fig. 10, Fig. 11), also seen at the delta scale (Fig. 2, Fig. 3). Pleistocene terraces and islands and thick undisturbed fine-grained floodplain deposits cannot be delineated solely from remote sensing and the compilation of existing geological borehole data, and require collection of additional data, e.g. resistivity profiles and OSL sediment dating.

Resistivity and gamma log profiles (Figs. 1B, Fig. 6, Fig. 7) resolved the sedimentary architecture of the alluvial system in great detail. Geophysical methods using resistivity have great potential for delineating both paleochannels (Chandra et al., 2019) and local, thick clay layers (Nath et al., 2010) in alluvial environments (Fig. 7). An important feature is also the Pleistocene sand island containing low As groundwater at Thuong Cat, which was identified from resistivity profiles and OSL dating (Fig. 10, Fig. 11). Such preserved Pleistocene aquifers may constitute an important local source of low As drinking water, especially if surrounded by floodplains with generally high As concentrations. The spatial extent of the Pleistocene island (Fig. 10) could not be delineated from the satellite image or topography, but was mapped using CVES and borehole data (Fig. 1B).

The interpreted resistivity threshold (30 ohmm) between fine-grained and coarse-grained sediments in the major part of the area is similar to the resistivity of <27 ohmm in thick clay layers in West Bengal, India (Nath et al., 2010). Resistivities in freshwater environments in the Red River delta show ranges of 15–25 ohmm in clay, 25–100 ohmm in silt and fine sand, and 100–200 ohmm in saturated coarse sand and gravel (Tran et al., 2012). The areas where the low resistive top layer has a thickness of >10 m have a high probability for the occurrence of high As groundwater (Nath et al., 2010). In the NE part of the study area, near Phung Town (Fig. 1B), high As concentrations occur in the central part of the low lying, undisturbed floodplain where the clay thickness exceeds 10 m (Fig. 8, Fig. 9, Fig. 10) and with little floodplain deposition since 4 ka BP (Funabiki et al., 2012b). Thinning of the clay layer, implying more flushing towards the edges of the clay filled lowland (Fig. 7D) results in lower As levels close to the Pleistocene sand island and old channel belts (Fig. 10).

The most recent channel along the Day River contains alternating sand and clay layers (Fig. 7B and E), which result in a high ‘swing intensity index’ that is correlated with a high As content in groundwater (Cao et al., 2018). The abundance of clay plugs in younger alluvial sediments (Figs. 7B, E and 8) compared to channel belt deposits with a burial age > 2 ka BP (Figs. 7A and 8) limits flushing of As from younger sediments and maintains high As concentrations (Donselaar et al., 2017).

### 5.3 Evolution of the alluvial system after 4 ka BP vs. the As distribution

The recent sedimentary architecture of the alluvial system is a product of sea level changes (Tanabe et al., 2006) and construction of dikes (Anh and Shannon, 2010). The low lying floodplain area hosts most of the Metal Age and Late Neolithic Age settlements (Funabiki et al., 2012b) which are located on a thick clay layer overlain by a thin younger clay layer deposited during the sea regression period starting at 4 ka BP (Fig. 4, Fig. 8, Fig. 9). The remaining settlements are located along the old channel belts along the SW delta boundary (Fig. 5) with likewise limited clay deposition after 4 ka BP (Fig. 8). The low sedimentation rate preserved the geomorphological paleoriver features along the SW margin of the floodplain and implies a limited supply of organic matter and more flushing, resulting in low As groundwater (Sø et al., 2018a). In floodplain areas where thick clay layers, which deposited during the sea level rise and high stand, were not exposed to erosion and channel belt deposition, the aquifers contain high groundwater As (Figs. 9A and 10). This is due to limited flushing (Sø et al., 2018a) and probably also a supply of reactive organic matter from the thick clay layers (Donselaar et al., 2017).

Channel belt migration during regression of the sea and prior to construction of the dikes was limited to the SW margin of the floodplain (Fig. 2, Fig. 9, Fig. 10). The recent Day River is the relict of a former major river in the delta (Funabiki et al., 2012b). During regression of the sea, vertical accretion in the western part of the Red River floodplain was substituted by lateral accretion (Funabiki et al., 2012b). Sediments of older channel belts were eroded and replaced with younger deposits, leaving only remnant islands of channel belt sand older than 4 ka (Fig. 9B). The area of old channel belt migration, outside of dikes, filled with relatively homogenous sand (Fig. 6) has low groundwater As (Fig. 10) due to the lack of thick overlying clay layers (Weinman et al., 2008). These conditions imply high extent of flushing (Sø et al., 2018a) and limited supply of organic matter (Kocar et al., 2014).

Most older deposits were eroded away in areas constrained by dikes. Inside the dikes, high As groundwater dominates in both Pleistocene and Holocene aquifers (Fig. 10). The migration of the active Red River channel and accretion of point bar deposits nowadays occur on a time scale of a couple of years (Figs. 4B and 9). It compares to the movement and sedimentation rate in the Mekong delta where a 1.5 × 5 km island of a point bar deposits was accreted within 31 years and reactive organic matter buried in the aquifer resulted in iron oxyhydroxides reduction and As enriched groundwater (Papacostas et al., 2008). The high As areas constrained by the dikes contain islands of the older channel belts with low As groundwater (Fig. 10). Groundwater As levels in these islands are probably a result of the same processes that govern As concentrations in channel belts along the SW floodplain margin. The remnants of the Mid Holocene deposits that could be a potential local source of low As groundwater within the dikes were delineated by combining remote sensing with geophysical and OSL data. However, the occurrence of low As groundwater in these islands needs to be confirmed by groundwater sampling, as they can be altered by changing hydrogeological and hydrochemical conditions (Kocar et al., 2014; Donselaar et al., 2017; Jakobsen et al., 2018).

Changes in groundwater As distribution during the geological evolution of the Red River floodplain over the last 6 ka were calculated using a two-dimensional (2D) reactive transport model by Jakobsen et al. (2018). The modeled groundwater As concentration increased with depth due to the decreasing number of recharged pore volumes (Jakobsen et al., 2018), consistent with the higher groundwater As level below the thick clay layer (Fig. 10). The model also predicted high As concentrations in stagnant zones below active river channels (Jakobsen et al., 2018). In general, the modeled groundwater As concentrations decreased with increasing sediment burial age and the number of recharged pore volumes, but remnants of high As groundwater in older deposits beneath former river channels were also predicted (Jakobsen et al., 2018). This may explain variations in the groundwater As level occurring even within the same geological unit (Fig. 10).

### 5.4 Remote sensing – Advantages and limitations for predicting the As groundwater level in rural areas

The delineation of various sedimentary environments based on satellite images has so far not been used in groundwater As predictions. Groundwater As levels cannot be predicted solely from satellite images. However, combining the delineation of sedimentary environments based on remote sensing and shallow geological data with existing groundwater As measurements enables predictions of groundwater As concentrations in rural areas, where no geological and chemical data exist (Fig. 2, Fig. 3).

The analysis of satellite images together with geophysical, geological and archeological data is also a useful tool in 3D modeling of floodplain geology (Fig. 8, Fig. 9). Previous work on the Red River delta used a geostatistical approach, employing compilations of 3D geology and soil features, to produce As probability maps (Winkel et al., 2011). It was found that both a model based on 3D geology and a model based only on surface soil features could predict the overall trends in the groundwater As concentration. However, while these methods do produce broad correlations between groundwater As content and geology or soil properties over larger areas, they are unable to more precisely identify localities with high As groundwater (Winkel et al., 2011; Lado et al., 2013; Sovann and Polya, 2014). A major problem appears to be the spatial scale of observations on which geology or soil maps are based. For example, distances between boreholes on which the 3D geological model is based (Winkel et al., 2011) require interpolation over distances that are significantly larger than the scale of spatial variability in the floodplain structure and in the groundwater As concentration. The small scale variability in sedimentary architecture of floodplains can be resolved in greater detail by using satellite images and geomorphology supported by scarce geological and archeological information (Fig. 2, Fig. 10), as shown in this study. Geophysical datasets can nowadays be obtained with moderate field efforts (Chandra et al., 2019). Including such data into modeling of floodplain geology captures sedimentary structures in more detail and could potentially lead to a higher precision in the prediction of As distribution.

The presented approach enables a more precise delineation of areas with an expected high As level, both at the delta and regional scale (Fig. 2, Fig. 10). However, it still cannot capture locally diverse groundwater As levels (as indicated by minimum and maximum values in Fig. 3, Fig. 11) due to the complexity of processes governing As distribution (Kocar et al., 2014; Donselaar et al., 2017; Jakobsen et al., 2018).

## 6 Conclusions

The study of sedimentary structures contained in a delta plain, based on remote sensing and ground based methods, can provide significant insight into the distribution of As in groundwaters of the floodplain on different levels. At the delta scale, the delineation of fluvial versus tidal- or wave-dominated sedimentary environments, using remote sensing, may give a first assessment of where low As groundwater can be expected as demonstrated in Fig. 2. Areas with the potentially highest As groundwater in the alluvial-dominated system can be delineated by mapping the youngest meander belts that remain clearly visible in satellite images. Such an approach will be useful in areas where no groundwater chemistry data is available.

At the local scale, the problem is always how to choose locations for boreholes where low As groundwater can be expected. As illustrated by the very high spatial variability in groundwater As content, this can be a major challenge. Detailed analysis of the sedimentary architecture of deposits, obtained by combining remote sensing, archeological, geophysical and borehole data, together with available groundwater As measurements offers the opportunity to predict the expected As concentration in groundwater of a given locality with more confidence, as it can delineate areas with similar sedimentology, stratigraphy and deposition history.

## Declaration of Competing Interest

The authors declare that they have no known competing financial interests or personal relationships that could have appeared to influence the work reported in this paper.
